# Occupational markers and pathology of the castrato singer Gaspare Pacchierotti (1740–1821)

**DOI:** 10.1038/srep28463

**Published:** 2016-06-28

**Authors:** Alberto Zanatta, Fabio Zampieri, Giuliano Scattolin, Maurizio Rippa Bonati

**Affiliations:** 1 Department of Cardiac, Thoracic and Vascular Sciences, Section of Medical Humanities, University of Padua Medical School, Italy; 2 Department of Medicine, University of Padua Medical School, Italy

## Abstract

Following the birth of modern opera in Italy in 1600, the demand for soprano voices grew up and the prepuberal castration was carried out to preserve the young male voice into adult life. Among the castrati, Gaspare Pacchierotti was probably one of the most famous. The remains of Pacchierotti were exhumed for the first time in 2013, for a research in the reconstruction of his biological profile, to understand the secrets behind his sublime voice and how the castration influenced the body. All the findings discovered, through anthropological and Computed Tomography analyses, are consistent both with the occupational markers of a singer and with the hormonal effects of castration. The erosion of cervical vertebrae, the insertion of respiratory muscles and muscles of the arms can be an effect of the bodily position and exercise during singing. The hormonal effect of castration were related to osteoporosis and to the disorders of spine.

Castration has been performed since centuries: from The Bible^1^ to the ancient Rome and China, until the 19th century, orchiectomy was made for different reasons from punishment for prisoners of war, to castration of mentally deficient men for eugenics laws^2^. In modern times, following the birth of modern opera in Italy in 1600, appeared a new men figure with single vocal parts consisting of arias and recitatives^3^. The demand for soprano voices grew up with the great contributions of Bach, Handel and others, so, from the end of the 16^th^ to the whole 18^th^ century, the prepuberal castration was carried out to preserve the young male voice into adult life^4^.

Among the castrati, Gaspare Pacchierotti (Fabriano, 1740 – Padua, 1821) was probably one of the most famous, at the point to be compared to current international pop stars. The remains of Pacchierotti were exhumed for the first time for research purposes in July 2013 (see supplementary information). The event was promoted by Medical Humanities’ research group of the University of Padua and Bepi Nalin, professor of music. We were interested in the biological profile reconstruction of Pacchierotti to understand the bodily secrets of his sublime voice.

Before Pacchierotti, only the body of Farinelli (1705–1782) (his real name was Carlo Maria Michelangelo Nicola Broschi), was studied in 2006, but the researchers found few remains not clearly attributable to him^5^. Pacchierotti’s body instead can be considered the first whole skeleton of a castrato ever studied. His remains were well preserved and could give new insights about his lifestyle and castration.

Information on Pacchierotti’s early life are lacking. We know that he was castrated before 12 years old, nevertheless he had many amorous relationship during his life.

Pacchierotti maintained the young male voice during the adult life. As Richard Edgcumbe, Earl of Mount Edgcumbe, described: “Pacchierotti’s voice was an extensive soprano, full and sweet in the highest degree: his powers of execution were great. […] Yet he was so thorough a musician that nothing came amiss to him; every style was to him equally easy, and he could sing, at first sight, all songs of the most opposite characters, not merely with the facility and correctness which a complete knowledge of music must give, but entering at once into the views of the composer, and giving them all the spirit and expression he had designed. Such was his genius in his embellishments and cadences, that their variety was inexhaustible”^6^.

Pacchierotti debuted when he was 19 in the *Teatro dei Nobili* in Perugia in a female role. In 1765, he was assumed in the St. Mark’s choir in Venice and he was singing in Innsbruck on the marriage of Peter Leopold of Habsburg-Lorraine and the Infanta Maria Luisa of Spain^7^.

Only one year later Pacchierotti could debut in theatre in Venice as opera singer and member of the choir, and his success brought him in the most famous theatre around Italy (Palermo, Naples, Milan, Padua, Genoa, and Turin). In Milan, he took the protagonist’s role at the inauguration of the *Teatro alla Scala* on 3 August 1778^7^.

Pacchierotti’s fame was not limited only to Italy, but spread around Europe: he went to London several times between 1779 and 1791, and he visited Marie Antoniette (1755–1793) in Paris in 1786. Once returned in Italy, Pacchierotti was called to the inauguration of the *Nuovo Teatro la Fenice* in Venice in 1792, where, one year later, he made his last operatic appearance. Finally he retired to Padua, first in the *Ca’ Farsetti* near Prato della Valle, formerly owned by the renewed humanist Pietro Bembo (1470–1547). Here he had lots of visits of famous artists, writers and politicians, like Carlo Goldoni (1707–1793), Vittorio Alfieri (1749–1803), Antonio Canova (1757–1822), Ugo Foscolo (1778–1827), Marie-Henri Beyle Stendhal (1783–1842), Gioachino Rossini (1792–1868), and even Napoleon Bonaparte (1769–1821), for whom Pacchierotti sang in the *Teatro Nuovo* in 1797^8^.

Then Pacchierotti decided to move in a Villa in the countryside of Padua, where he died by dropsy at the age of 81 and was buried inside a little chapel, next to the villa, in a tomb under the floor level (see supplementary information). As already mentioned, Pacchierotti was exhumed for the first time by our research group on 2^nd^ July 2013.

## Methods

**Figure 1:**
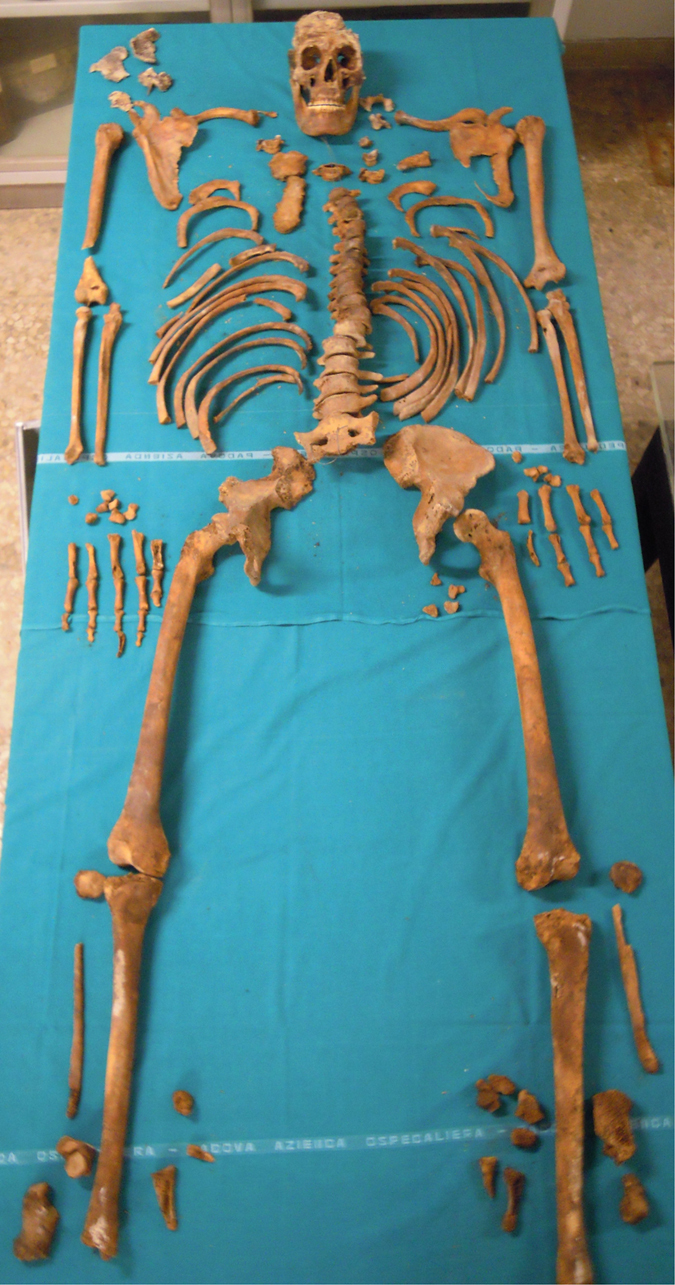
Pacchierotti’s skeleton.

Once opened the headstone of the grave, the skeleton of Pacchierotti was observed to be laid down with the head pointed towards east, just under the tomb’s opening. All the remains were quite well preserved, but, because of the presence of plaster pieces and water infiltration, in absence of any protective structures, part of the skull was damaged (Fig. 1). After the recovery of all the remains, the skeleton was carried in the laboratory of the Museum of Pathological Anatomy of Padua University for analysis.

We made different anthropological measurements on the remains to understand Pacchierotti’s biological profile and how the castration influenced his body. We performed Computed Tomography (CT) analysis on all the skeleton and X-ray microtomography (MM) on some bones’ samples. We used a 64-section scanner CT (Somatom Sensation 64, Siemens Medical Solutions, Erlangen, Germany) in the Radiology Department of Padua University. The appropriate settings were adjusted as to not exceed the thermal capability of the x-ray tube (120 kV, 100 mAs). It was selected the minimum scanning thickness allowed by the CT unit (0.6 mm). A Multi-modality workplace (Syngo Siemens) was used to reformat the data set in the coronal and sagittal planes. Volume rendered and surface-shaded-display three-dimensional models as well as three-dimensional animated virtual “fly-through” were created to facilitate analysis.

## Results

**Figure 2:**
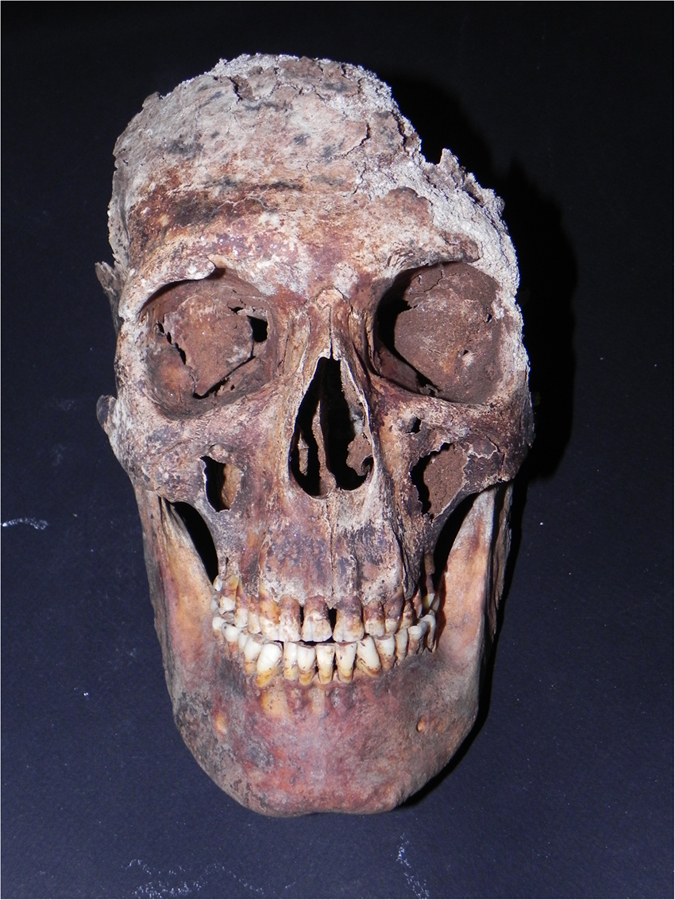
Pacchierotti’s skull.

From a first morphological analysis, the skeleton revealed typical male features, because of the indicator of sex in hips^9^, femurs^10^, mandible and skull^11^. Unfortunately, the skull was fragmented and most of the neurocranium was missing, except for the frontal and the right temporal bones (Fig. 2). By consequence, it was impossible to measure the level of fusion of the cranial sutures to calculate the age. At any case, many others skeletal age markers were preserved: the occlusal surfaces of the teeth were worn and characteristic of a man in advanced age^12^,^13^. Also the auricular surface of the ilium was consistent with old age^14^, because it was porous with areas of erosion and its edges were irregular with presence of osteophytes. Moreover, the borders of the sternal end of the ribs were extended, ragged and thin with a porous and irregular surface, typical of a senile man^15^.

**Figure 3:**
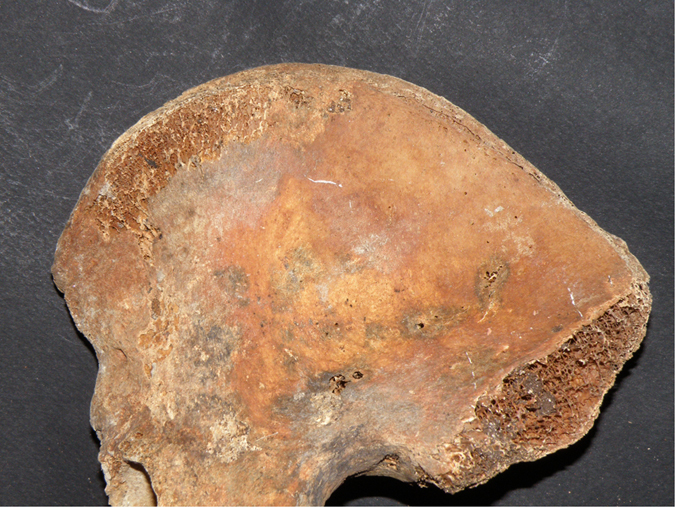
Persistence of epiphyseal line in the iliac crest.

Even if all these skeletal markers confirmed the advanced age at death of Pacchierotti (81 years old), the epiphyseal lines on the iliac crests were visible (Fig. 3). These lines usually are fused at 23 years old^16^ and no more traces can be found in 90–100% of over 35 years old males^17^,^18^.

**Figure 4:**
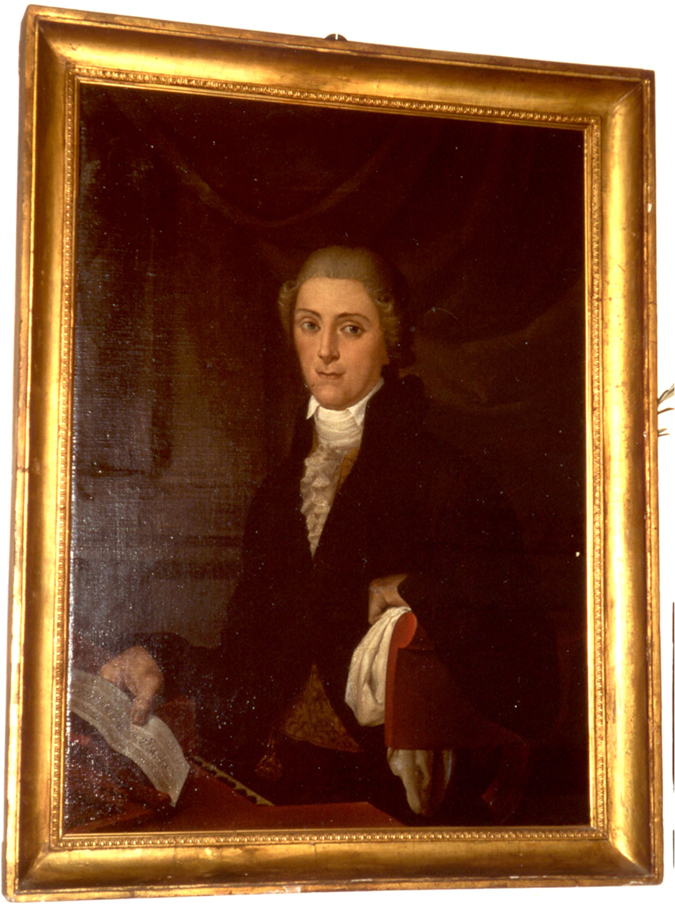
Portrait of Pacchierotti.

Castrati were usually tall, with a large barrel-shaped chest, infantile larynx, long, spindly legs^3^. Pacchierotti’s bones confirmed these characteristics, in particular the height, that was estimated measuring femurs, tibiae and humeri lenght^19^,^20^ and gave a value of around 191 cm. However, “Pacchierotti was not beautiful at all, rather really ugly. He was thin and gangly and he had a strange nose. […] That’s why he didn’t love having portraits”^7^ (Fig. 4). Pacchierotti’s face was long with narrow orbits and a large and wide nasal cavity. The internal palate was short and narrow as the alveolar arch.

**Figure 5:**
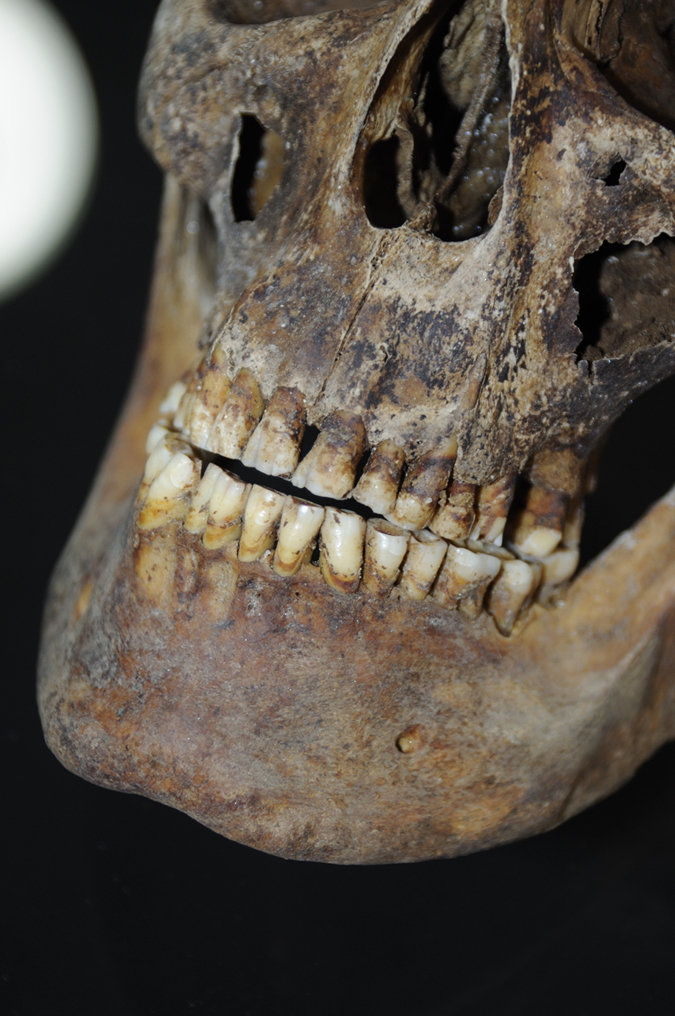
Dental condition of Pacchierotti.

The dental condition of Pacchierotti is very interesting (Fig. 5). The singer presented a skeletal Class III with moderate dental compensation that took him to an occlusion “head to head”. The periodontal condition is excellent, with only a mesial intrabony pocket greater than 3 mm in 36 without any other involvement. The absence of 26 is clear, with the presence of a post-extraction site completely cured and a healed bone. In addition, the 16 is absent probably due to a recent tooth extraction, at least 6 weeks before death.

The articular condyles of the mandibula are flattened with an initial osteophytes hint on the left condyle. The mandibular angle is pronounced, as some portraits of Pacchierotti confirm (Fig. 4). There was an extremely advanced dental erosion due to bruxism, that was probably caused by psychic distress from compulsion as it happens in prisoners or people forced to do something^21^. There was also enamel hypoplasia evident on 43, 33, 34, 35, a probable sign of trauma occurred at a young age that could be related to the castration. In general, the teeth were well preserved compared to the age of Pacchierotti. It remains impressive the way Pacchierotti had care of his dental hygiene; evidently, he considered his mouth a real working tool to keep in the best possible way, also using a sort of dental floss and some abrasive substances, as minced sage and salt, to whiten the teeth. At the same time, there was an underdevelopment of the maxilla with moderate mandibular prognathism. The tooth wear could be caused by stressing parafunctional habits or from food containing abrasive material in use at the time, as for example could be in the food flour, or even could be the result of traumatic tooth brushing in horizontal scrubbing movements.

**Figure 6:**
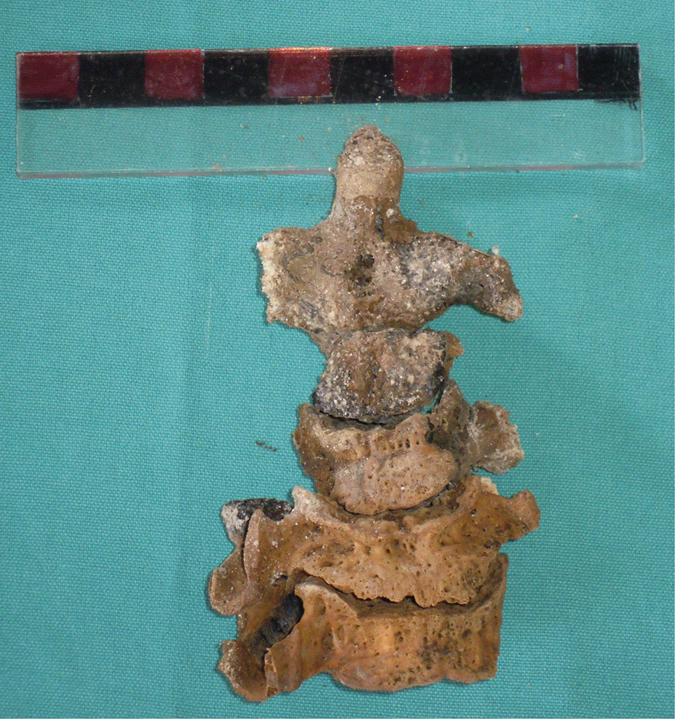
Strong erosion of the cervical vertebrae.

Pacchierotti’s cervical vertebrae were all strongly eroded with signs of osteophytic lipping in the body, because of osteoporosis and of continuous movements of head and neck during singing exercises (Fig. 6). These conditions can be considered as an occupational marker for the singers, as it was confirmed by Pacchierotti’s vertebral conditions. These findings are very significant, because it is the first time that they can be observed in the remains of a castrato.

Another important marker found in Pacchierotti’s remains was the insertion of three important respiratory muscles on the second ribs, the scalenus posterior, which elevates the second rib, the serratus anterior, which can lift the ribs and assist in respiration, and the serratus posterior superior, that elevates second to fifth ribs and aids deep inspiration.

Both scapulae had a marked infraglenoid tubercle due to a strong insertion of the long head of the triceps brachii muscle, which acts on the shoulder joint and is involved in retroversion and adduction of the arm. Probably Pacchierotti was using a lot his arms to act during his performances.

Pacchierotti’s lumbar vertebrae presented important osteophytes as a sign both of the age and the degeneration in the spine that could cause him lower back pain, and compression of the sciatic nerve.

**Figure 7:**
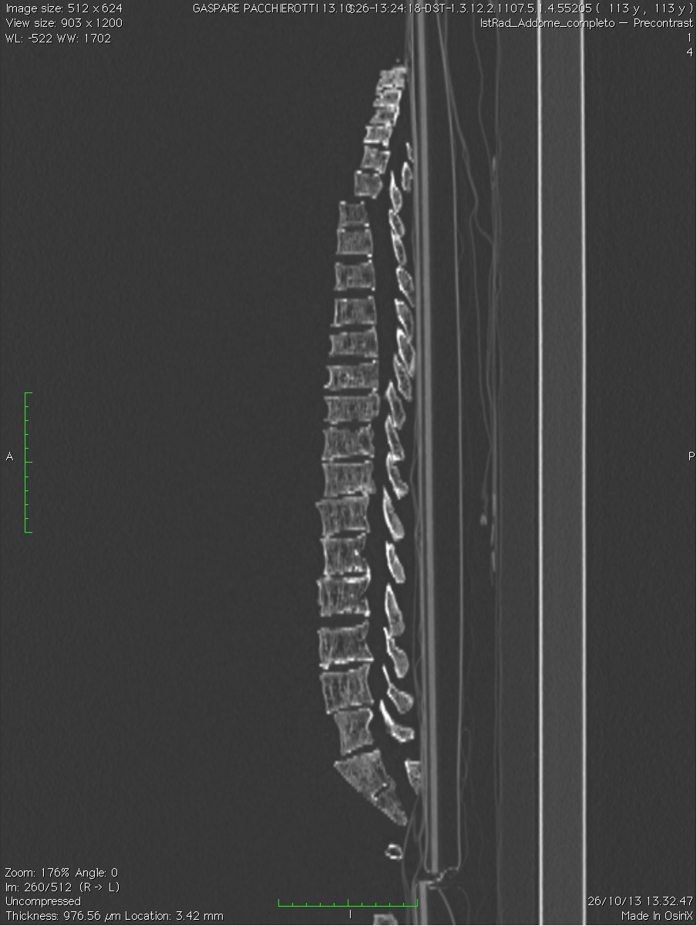
CT scan of the spine of Pacchierotti.

To improve our analysis, we performed a Computed Tomography on the whole skeleton and X-ray MM on some bones’ samples. The CT scan pointed out a general calcareous atrophy with multiple cribrosus bones in the vertebral walls and other problems on the spine: the cervical vertebrae were strongly eroded; there was an anterior wedge-shaped vertebra (T7), an anterior vertebral wall fracture in both L1 and L2 that can be due to osteoporosis; there were also several osteophytes between L3 and L4, and a Schmorl’s nodes in the vertebral body endplate of L4 (Fig. 7).

All the most salient anatomical features associated with castration and professional singing are listed in Table 1.

## Discussion

The anthropological analysis of Paccherotti’s remains underlined the presence of many characteristics related to castration: high stature, open epiphyseal lines in the hips, lower cortical bone density related to vertebral fractures. That “castrati” were particularly tall has been known since antiquity^22^, even Aristotle observed that “all animals, if operated on when young, become bigger than their unmutilated fellows”^23^. At the beginning of the XX century, the Skoptzy, a Christian sect practicing male castration, were measured and they appeared to be taller than their peers^24^. The persistence of the epiphyseal lines was also found in the iliac crest of another castrato singer, Farinelli^5^, and can be considered as a typical marker in young age castrati (Fig. 3). Tandler and Grosz at the beginning of the XX century described the failure of closure of the epiphyses in the skeleton of eunuchs^25^,^26^. Sex hormones preserve bones, at least in part, by regulating the development and death (apoptosis) of osteoclasts and osteoblasts; this adjustment is permitted by the modification of cytokine production and of the sensitivity of bone marrow progenitor cells to cytokines themselves. For example, the production of interleukin-6 (IL-6) by the osteoblasts is inhibited by estrogen and androgen. After menopause or following castration in men, bone loss increases by 10 times, largely due to overproduction of IL-6. The estrogen deficiency seems to delay osteoclasts apoptosis, while promoting the osteoblast: it follows an imbalance between reabsorption and bone formation. In addition, the delay of osteoclasts apoptosis could be responsible for an increase in the depth of the cavities of resorption, and, consequently, of the trabecular perforation resulting from estrogen deficiency^27^. It is known the existence of a hormonal crosstalk between bone and testis; it includes testosterone and vitamin D that, released by the testes, affect bone remodeling and the bone-derived hormone osteocalcin that may control testosterone production^28^.

**Figure 8:**
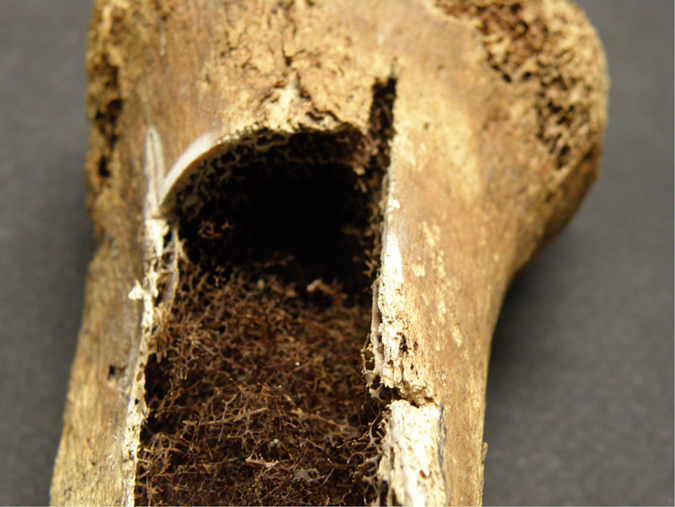
Tibia with a decrease of cortical bone density.

Characteristics of castration’s bone loss are vertebral fractures, as revealed with the CT scan (L1 and L2), but also the micro-CT found a decrease in cortical bone density, especially in long bones as tibiae and humeri (Fig. 8). The CT scan demonstrates a diffuse osteoporosis affecting both the cortical and the cancellous bone. This is different from senile osteoporosis, where remnants of a normal bone would be seen as microfractures as well as repairs. In fact, X-ray’s results are remarkable because of the relative absence of osteoporotic microfractures. This suggests a long-standing adaptation.

Bone mineral density progressively decreases after castration, particularly in the first few years, causing osteoporosis^29^. As in Pacchierotti, a study by Stepan *et al*., found a progressive loss of the lumbar bone density after orchiectomy^29^. Kock, studying the men of Skoptzy sect with X-ray, found that kyphosis was common because of osteoporosis^30^. The same kyphosis was observed by Wagenseil in 20 out of the 31 Chinese eunuchs^31^.

Osteoporosis is clearly proved also in experimental rats. One-year-old castrated rats developed pronounced femoral osteoporosis 4 months after castration^32^. Finally, orchiectomy for prostate cancer is frequently followed by severe osteoporosis^33^.

In the study of Farinelli^5^, it was found, in a fragment of the frontal bone, a severe hyperostosis frontalis interna (HFI) that the authors related to the castration of the singer, but there were no signs of this pathology in the skull of Pacchierotti. Since the HFI can have different aetiologies^34^, we can assume that HFI is not always correlated with castration. Moreover, Kock, in the already mentionedstudy on Skoptzy, reported that in all of them was evident the thinning of the bones of the skull^30^.

Pacchierotti’s activity as singer determined some changes in his body and bones. We discovered modifications in the insertion of three important respiratory muscles: scalenus posterior, serratus anterior and serratus posterior superior. Pettersen in different studies found equivalent modifications of scalenus and serratus in professional opera singers by measuring the electromyographic activity^35^,^36^.

Pacchierotti’s cervical vertebrae were all strongly eroded, as demonstrated by the osteophytic lipping revealed from the CT scan. According to Miller *et al*.^37^, who measured by Magnetic resonance imaging (MRI) pitch-related adjustments in ten healthy volunteers, high note humming was accompanied by increased craniocervical angles. In singing, the position of the atlas with respect to the true vertical (P < 0.001), the axis (P < 0.001) and the C4 vertebra both with respect to the horizontal (P < 0.001), and the axis with respect to the cranium (P < 0.001), were all significantly different to those at rest^38^. This prolonged condition in Pacchierotti, combined with his osteoporosis, can account for the eroded states of his cervical vertebrae. Di Carlo carried out a systematic X-ray study of cervical spine of three populations of subjects (professional singers, beginning singers and non-singers), which showed deformations of cervical spine after many years of intensive singing^39^.

According to research in phoniatrics^40^,^41^, the ideal posture of the neck in a singer is with the back of the neck elongated compared with the position of the shoulders, so that the rotation of the neck-head is not limited. This technique also permits to avoid passive tilting, lifting or stretching of the laryngeal box, allowing that freedom of management of the glottal-upper glottal system that promotes a greater ease of development of harmonics. An incorrect posture at this level has an influence not only on the stamp, but also on the respiratory dynamics. A correct postural attitude requires the cervical spine to be maintained in an erect position, in nuchal elongation, avoiding the lordosis, the extension and lifting of the jaw. This particular posture determined the progressive erosion of Pacchiarotti cervical vertebrae. A correct postural alignment of the head and neck, in fact, is a necessary element in the optimization of voice production^38^. Finally, the great bodily size of Pacchiarotti, in particular of his chest, was positively associated with the power of his voice, described as an “extensive soprano”^41^.

In conclusion, this research describes, for the first time, the occupational markers and the hormonal effect of castration on a whole skeleton of a castrato singer integrating the knowledge about castration in men and animals and its effects on the skeleton. It provides also an enlightening description of the occupational markers of professional singers.

“Today we can but guess what the great singers of the past can have sounded like; but one might hazard a guess that of all the castrati, could we hear them, Pacchierotti would please us most”^42^.

## Additional Information

**How to cite this article**: Zanatta, A. *et al*. Occupational markers and pathology of the castrato singer Gaspare Pacchierotti (1740–1821). *Sci. Rep.* **6**, 28463; doi: 10.1038/srep28463 (2016).

## Supplementary Material

Supplementary Information

## Figures and Tables

**Table 1:** Anatomical features associated with professional singing and castration in Pacchierotti.

Castration	Occupational markers	Castration + Markers
Long long-bones	Strong insertion of scalenus posterior muscle	Strong erosion of cervical vertebrae
Enamel hypoplasia	Strong insertion of serratus anterior muscle	
Bruxism	Strong insertion of serratus posterior superior muscle	
Epiphyseal line on the iliac crests are visible	Strong insertion of the long head of the triceps brachii muscle	
Decrease of cortical bone density		
