# Supplementary material for: Occupational markers and pathology of the castrato singer Gaspare Pacchierotti (1740–1821)
Source: Sci Rep. 2016 Jun 28;6:28463. doi: 10.1038/srep28463 (PMC4923859; doi:10.1038/srep28463)
Supplement: Supplementary Information [file srep28463-s1.pdf]

Supplementary information

Occupational markers and pathology of the castrato singer Gaspare Pacchierotti (1740-1821).

Alberto Zanatta,[1]* Maurizio Rippa Bonati,[1] Giuliano Scattolin,[2] Fabio Zampieri[1]

[1] Department of Cardiac, Thoracic and Vascular Sciences, Section of Medical Humanities, University of Padua Medical School.

[2] Department of Medicine, University of Padua Medical School.

* Corresponding author: University of Padua Medical School, Department of Cardiac, Thoracic and Vascular Sciences, Section of Medical Humanities. via Aristide Gabelli, 61 - 35121 Padova (Italy), Tel: 049/8272269, E-mail: alberto.zanatta.1@unipd.it

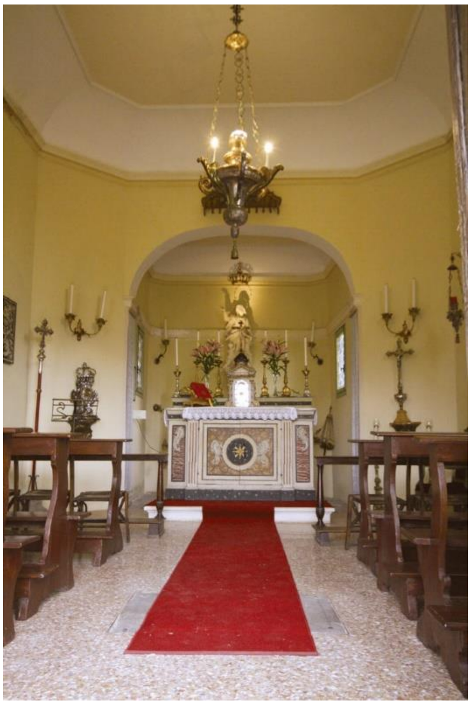

The chapel where there is the tomb of Pacchierotti.

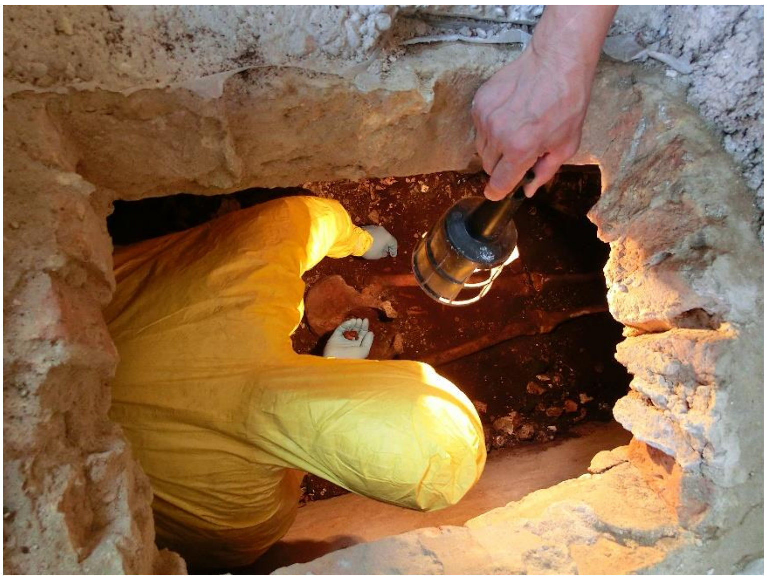

Anthropologist recovering the skeletal remains.
